# Global Epidemiology of Vector-Borne Parasitic Diseases: Burden, Trends, Disparities, and Forecasts (1990–2036)

**DOI:** 10.3390/pathogens14090844

**Published:** 2025-08-25

**Authors:** Cun-Chen Wang, Wei-Xian Zhang, Yong He, Jia-Hua Liu, Chang-Shan Ju, Qi-Long Wu, Fang-Hang He, Cheng-Sheng Peng, Mao Zhang, Sheng-Qun Deng

**Affiliations:** 1 Department of Pathogen Biology, Anhui Province Key Laboratory of Zoonoses, The Provincial Key Laboratory of Zoonoses of High Institutions in Anhui, School of Basic Medical Sciences, Anhui Medical University, Hefei 230032, China; wangcunchen@stu.ahmu.edu.cn (C.-C.W.); zhangweixian@stu.ahmu.edu.cn (W.-X.Z.); 2345010058@stu.ahmu.edu.cn (Y.H.); 18646709766@163.com (J.-H.L.); juchangshan2025@163.com (C.-S.J.); 2313270007@stu.ahmu.edu.cn (Q.-L.W.); 2314010014@stu.ahmu.edu.cn (F.-H.H.); 2214011004@stu.ahmu.edu.cn (C.-S.P.); 2 Department of Pathology, The Second Affiliated Hospital of Anhui Medical University, Hefei 230601, China

**Keywords:** vector-borne parasitic diseases, Global Burden of Diseases 2021, Socio-demographic Index, disability-adjusted life years

## Abstract

Vector-borne parasitic diseases (VBPDs), including malaria, schistosomiasis, leishmaniasis, Chagas disease, African trypanosomiasis, lymphatic filariasis, and onchocerciasis, impose a significant global health burden. This study analyzes the global disease burden of VBPDs from 1990 to 2021 using Global Burden of Disease (GBD) 2021 data and projects trends to 2036. Metrics include prevalence, deaths, disability-adjusted life years (DALYs), and age-standardized rates (ASRs) across regions, sexes, age groups, and Socio-demographic Index (SDI) levels. Key findings reveal persistent disparities: malaria dominated the burden (42% of cases, 96.5% of deaths), disproportionately affecting sub-Saharan Africa. Schistosomiasis ranked second in prevalence (36.5%). While African trypanosomiasis, Chagas disease, lymphatic filariasis, and onchocerciasis declined significantly, leishmaniasis showed rising prevalence (EAPC = 0.713). Low-SDI regions bore the highest burden, linked to environmental, socioeconomic, and healthcare access challenges. Males exhibited greater DALY burdens than females, attributed to occupational exposure. Age disparities were evident: children under five faced high malaria mortality and leishmaniasis DALY peaks, while older adults experienced complications from diseases like Chagas and schistosomiasis. ARIMA modeling forecasts divergent trends: lymphatic filariasis prevalence nears elimination by 2029, but leishmaniasis burden rises across all metrics. Despite overall progress, VBPDs remain critical public health threats, exacerbated by climate change, drug resistance, and uneven resource distribution. Targeted interventions are urgently needed, prioritizing vector control in endemic areas, enhanced surveillance for leishmaniasis, gender- and age-specific strategies, and optimized resource allocation in low-SDI regions. This analysis provides a foundation for evidence-based policy and precision public health efforts to achieve elimination targets and advance global health equity.

## 1. Introduction

Vector-borne diseases refer to a category of illnesses transmitted through specific biological vectors. The prevalence of these diseases is influenced by natural, economic, social, and other factors, accounting for more than 17% of all infectious diseases and forming a considerable challenge to the health of populations globally [1]. Among these diseases, vector-borne diseases caused by parasites are termed vector-borne parasitic diseases (VBPDs). The predominant diseases in this category encompass malaria, lymphatic filariasis, leishmaniasis, African trypanosomiasis, Chagas disease, onchocerciasis, and schistosomiasis [1,2]. Malaria, caused by *Plasmodium* parasites and transmitted via *Anopheles* mosquitoes, remains a major international health concern. While World Health Organization (WHO) data from 2023 indicate a marginal decline in malaria-related mortality, the incidence rate has exhibited a slight upward trend due to increased international travel and immigration, underscoring its persistent threat to global health systems [3,4,5]. Lymphatic filariasis (elephantiasis) is etiologically linked to filarial nematodes *Wuchereria bancrofti*, *Brugia malayi*, and *Brugia timori*, with mosquitoes serving as the principal vector. Although the disease is typically not fatal, it damages the lymphatic system and leads to abnormal enlargement of body parts, which results in its status as the second major contributor to global disability [6,7]. According to the WHO, over 657 million people in 39 countries or regions around the world remain at risk of the disease [8]. Leishmaniasis, a parasitic disease induced by *Leishmania* species, is disseminated through dermal exposure to infected female sandfly vectors during blood feeding. The disease affects approximately 700,000 to 1 million people yearly, among whom visceral leishmaniasis constitutes the most critical presentation. Malnutrition, displacement, inadequate housing conditions, weakened immune systems, and financial hardship constitute major risk factors [9]. Chagas disease is caused by *Trypanosoma cruzi* and is transmitted primarily through contact with the feces or urine of infected triatomine bugs. Its global prevalence is rising, and the burden falls mainly on Latin America. This disease is a result of complex health issues, and although a cure is possible, urbanization, globalization, and emerging transmission routes have made it a global public health challenge [10,11]. African trypanosomiasis, colloquially termed sleeping sickness, is due to infection with *Trypanosoma brucei* and vectored by the tsetse fly. It is concentrated in certain areas, predominantly in sub-Saharan Africa. Left untreated, the condition is typically life-threatening. In recent decades, both the incidence and distribution of this disease have decreased [12,13]. The cause of schistosomiasis is parasitic worms of the genus *Schistosoma*. This disease spreads primarily when skin contacts freshwater harboring infectious cercariae, with Asia, Africa, and Latin America being its primary endemic regions. The disease has a high incidence, with approximately 1 billion people globally at risk. Owing to the complexity of the transmission chain, high rates of reinfection, potential drug resistance, limitations in diagnostic techniques, and socioeconomic barriers, schistosomiasis significantly strains worldwide health resources and is a major driver of poverty and reversion to poverty [14,15]. Onchocerciasis, or river blindness, is a parasitic infection resulting from *Onchocerca volvulus* and transmitted via black fly bites. The global burden of this disease is concentrated in sub-Saharan Africa. Affecting both the eyes and skin, it can, in the most severe form, result in visual impairment or irreversible blindness [16]. While mass drug administration has effectively controlled the disease, it has not been completely eradicated and continues to persist in many endemic regions of Africa [17].

VBPDs impose a significant burden on individuals, families, and national economies, especially in under-resourced, impoverished areas experiencing health service shortages. Vector control remains a critical measure for addressing VBPDs. Although many countries have successfully curbed the transmission of these diseases through vector control strategies, such approaches still face significant challenges. Insecticide resistance developing and spreading in key vectors compromises the effectiveness of critical interventions, such as insecticide-treated nets (ITNs), long-lasting insecticidal nets (LLINs), and indoor residual spraying (IRS) [18,19]. Furthermore, there is growing evidence of adaptive changes in vector behavior, such as increased outdoor and early-evening biting activity, particularly noted in malaria vectors like *Anopheles* mosquitoes [20]. This behavioral shift reduces the protective impact of interventions primarily targeting indoor-biting and nighttime-biting vectors. Operational hurdles remain, including maintaining intervention coverage and access, ensuring effective community engagement and adequate funding, and establishing robust surveillance to track vectors and disease transmission, particularly in remote or conflict-affected areas [1,21]. Addressing these multifaceted limitations requires continuous innovation in vector control tools and strategies, alongside strengthened health systems. To better utilize medical resources, formulate response measures, and increase public awareness of personal prevention, it is essential to assess the global, national, and regional burdens of VBPDs and their evolving trends. Accordingly, this research employs data from the Global Burden of Disease (GBD) 2021 database to analyze trends in the prevalence, deaths and disability-adjusted life years (DALYs) of VBPDs across geographic regions, age groups, sexes, and Socio-demographic Index (SDI) levels. It evaluates the distribution of VBPDs among populations and regional characteristics and forecasts the global disease burden for the next 15 years. These findings not only highlight the persistent threat that VBPDs pose to human health but also provide a foundation for the precise design of targeted intervention measures and the scientific allocation of community health assets.

## 2. Methods

### 2.1. Data Source and Extraction

The GBD 2021 quantifies the burden of 371 diseases and injuries across 204 countries and territories worldwide from 1990 to 2021, categorized by country or region, year, sex, and age group. Data on these VBPDs can be accessed through the Global Health Data Exchange (GHDx) Results Tool (http://ghdx.healthdata.org/gbd-results-tool, accessed on 6 December 2024) and can be obtained through a web browser on its official website. The SDI is a core metric in the GBD 2021 framework, capturing the socio-economic factors that shape regional health outcomes. It is calculated using the under-25 fertility rate, the average education level of the population aged 15 and older, and lag-distributed per capita income [22]. In GBD 2021, SDI values range from 0.00 to 1.00 and classifies locations into five development levels: low (<0.46), low-middle (0.46–0.60), middle (0.61–0.69), high-middle (0.70–0.81), and high (>0.81) [23,24].

The data used in this study, including prevalence, deaths and DALYs for VBPDs, were extracted from GBD 2021. The detailed search conditions were as follows: indicators (prevalence, deaths, DALYs), measures (number, rate), causes (malaria, schistosomiasis, African trypanosomiasis, Chagas disease, lymphatic filariasis, onchocerciasis, leishmaniasis), regions (global, different SDI regions, 21 GBD regions), ages (all ages, age-standardized, <5 years, 5–9 years, 5–14 years, 10–14 years, 15–19 years, 15–49 years, 20–24 years, 25–29 years, 30–34 years, 35–39 years, 40–44 years, 45–49 years, 50–54 years, 50–74 years, 55–59 years, 60–64 years, 65–69 years, 70–74 years, 75 years and older, 75–79 years, 80–84 years, 85–89 years, 90–94 years, 95 years and older), genders (both, male, female), and years (1990–2021, and each year from 1990–2021).

### 2.2. Definition of the VBPDs

Vector-borne diseases, as defined by the WHO, refer to illnesses transmitted by vectors. Vectors are organisms capable of transmitting pathogens (such as bacteria, viruses, or parasites) from infected individuals or animals to humans or other animals. Among vector-borne diseases, those caused by parasites include malaria, leishmaniasis, Chagas disease, African trypanosomiasis, schistosomiasis, lymphatic filariasis, and onchocerciasis. These diseases constitute a major global health challenge. With the exception of malaria, the other six diseases are classified by the WHO and the GBD 2021 as neglected tropical diseases. Owing to their prevalence in impoverished and remote areas and their complex epidemiology, controlling these diseases presents numerous challenges. On the basis of these findings, we operationalize the term “vector-borne parasitic diseases (VBPDs)” to encompass malaria, leishmaniasis, Chagas disease, African trypanosomiasis, schistosomiasis, lymphatic filariasis, and onchocerciasis.

### 2.3. Statistical Analysis

We employed a linear regression model and natural log transformation to fit the data, assuming a linear relationship between the natural logarithm of the age-standardized rate (ASR) (Y) and the calendar year (X) with random deviation (ε), as expressed by the equation Y = α + βX + ε, where β represents the direction and magnitude of ASR change. The estimated annual percentage change (EAPC) and its corresponding 95% confidence interval (CI) were used to assess the trends in the age-standardized prevalence rate (ASPR), age-standardized death rate (ASDR), and age-standardized DALY rates for VBPDs from 1990 to 2021. The formula for EAPC is EAPC = 100 × (exp(β) − 1). The linear model provides a 95% CI. If the EAPC is positive and the lower limit of its CI is also positive, it indicates an upward trend in ASR; if the EAPC is negative and the upper limit of its CI is also negative, it indicates a downward trend in ASR.

This study employs the autoregressive integrated moving average (ARIMA) model to predict the global disease burden of VBPDs over the coming 15 years. The ARIMA model is a commonly used statistical approach for time series analysis and prediction, denoted as ARIMA (p, d, q), where p represents the order of the autoregressive terms, d indicates the number of differences required for stationarity, and q signifies the order of the moving average terms [14,25]. By analyzing historical data, this study predicts the ASPR, ASDR, and age-standardized DALY rates for VBPDs globally from 2022 to 2036. The optimal ARIMA model was selected using the Akaike Information Criterion (AIC) and Bayesian Information Criterion (BIC) to forecast the disease burden trend of VBPDs from 2022 to 2036. The Ljung–Box test confirmed that the residuals of the selected model followed an independent normal distribution. All the statistical analyses were performed via the R program (version 4.4.2).

## 3. Results

### 3.1. Global Burden of VBPDs

**Figure 1:**
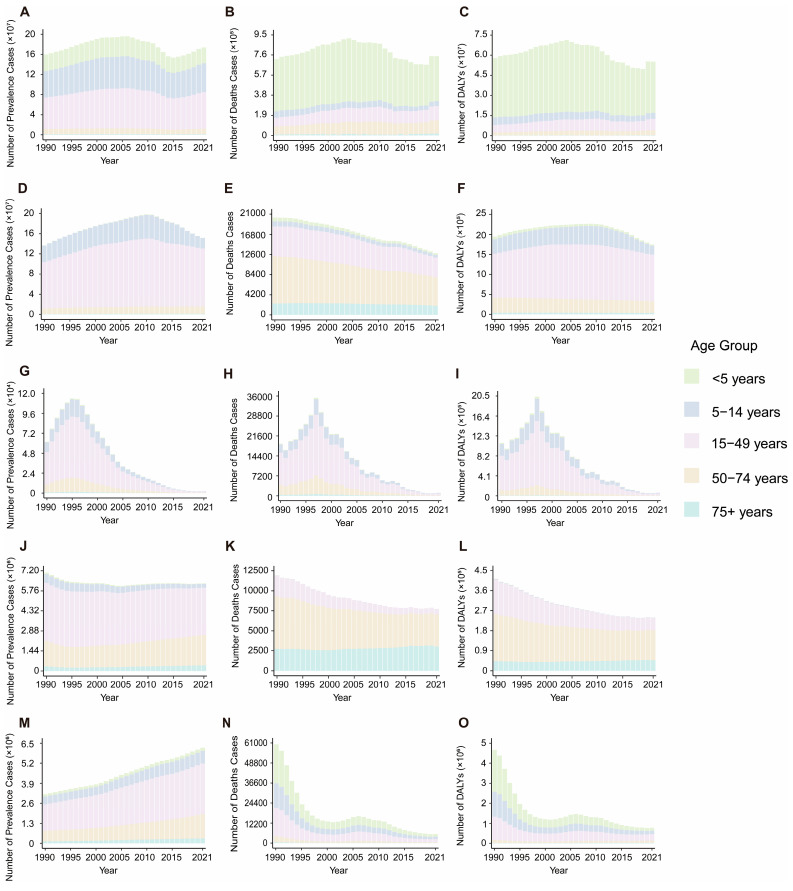
Global age distribution and trends in the number of prevalence, deaths, and DALYs from 1990 to 2021. (**A**–**C**), malaria. (**D**–**F**), schistosomiasis. (**G**–**I**), African trypanosomiasis. (**J**–**L**), Chagas disease. (**M**–**O**), leishmaniasis. DALYs, disability-adjusted life years.

**Figure 2:**
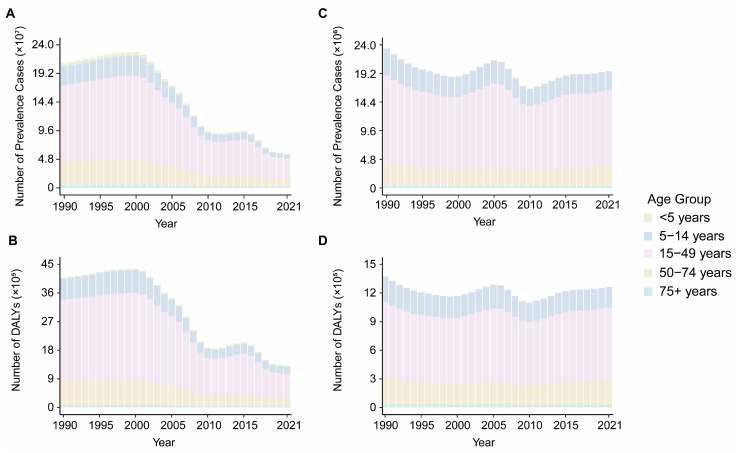
Global age distribution and trends in the number of prevalence and DALYs from 1990 to 2021. (**A**,**B**), lymphatic filariasis. (**C**,**D**), onchocerciasis. DALYs, disability-adjusted life years.

Globally, VBPDs continue to impose significant health burdens. From 1990 to 2021, the global case numbers of African trypanosomiasis, Chagas disease, lymphatic filariasis, and onchocerciasis decreased, whereas the number of cases of leishmaniasis, malaria, and schistosomiasis increased (Figure 1 and Figure 2). Malaria remains the most impactful disease, with an estimated 173.88 million prevalence cases and 748,131.24 deaths worldwide in 2021. The corresponding ASPR and ASDR were 23,367.935 and 105.072 per 1,000,000 population, respectively. The global DALY burden reached approximately 55.17 million, with an age-standardized DALY rate of 805.998 per 100,000 population (Figure 1A,B, Tables S1–S3). Schistosomiasis ranked second in prevalence, with 151.38 million cases and an ASPR of 19,142.985 per 1,000,000 population (Figure 1D, Table S1). Notably, relative to 1990, the global ASPR for leishmaniasis slightly increased, with an estimated annual percentage change (EAPC) of 0.713, whereas the ASDR and age-standardized DALY rate declined. With the exception of leishmaniasis, the ASPR, ASDR, and age-standardized DALY rates for the other six VBPDs decreased (Tables S1–S3). In contrast, the burden of African trypanosomiasis has significantly diminished, with 2,367.93 cases, 1,055.32 deaths, and 61,962.48 DALYs reported in 2021. In the years 1990 through 2021, the ASPR, ASDR, and age-standardized DALY rates for African trypanosomiasis all experienced substantial declines (Figure 1G–I, Tables S1–S3).

### 3.2. Regional Burden Disparities in the VBPDs

**Figure 3:**
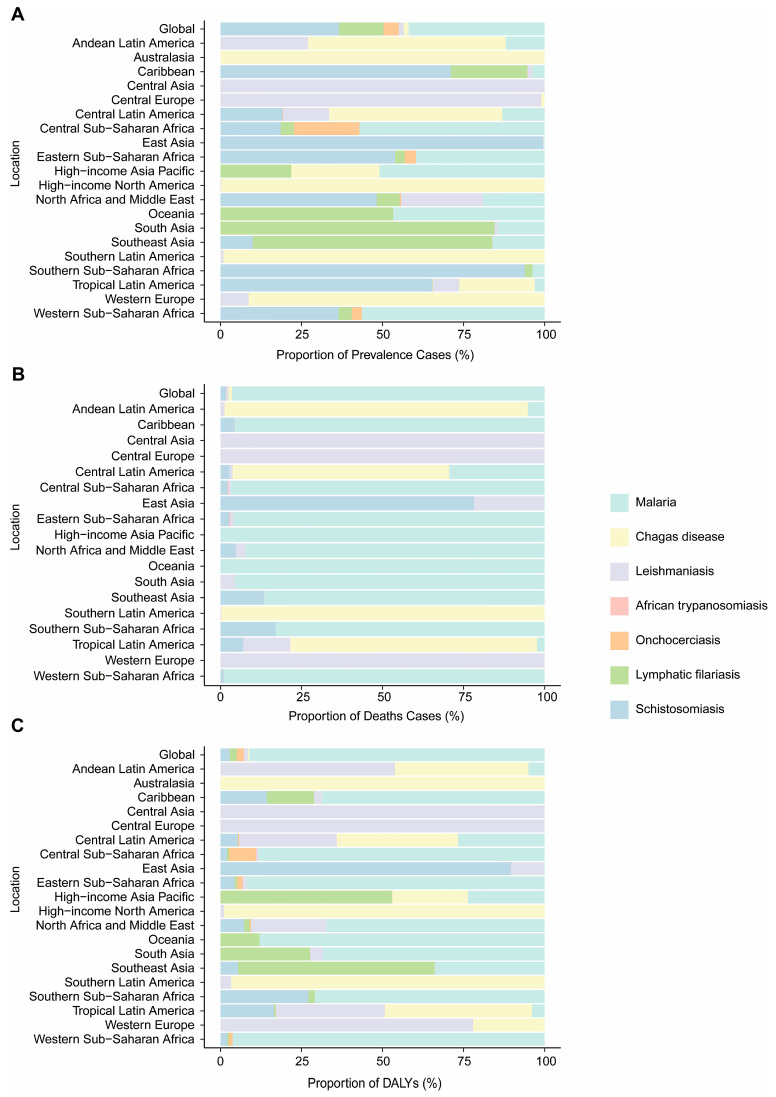
Proportion of prevalence (**A**), deaths (**B**), and DALYs (**C**) for VBPDs globally and by region in 2021. VBPDs, vector-borne parasitic diseases; DALYs, disability-adjusted life years.

In 2021, the global burden of VBPDs varied significantly (Figure 3, Tables S4–S6). Globally, malaria accounted for the largest proportion of total cases (42.0%), followed by schistosomiasis (36.5%), whereas African trypanosomiasis represented the smallest share (0.00057%) (Figure 3A). Malaria dominated global deaths (96.5%) (Figure 3B) and similarly constituted the vast majority of DALYs (91.1%) (Figure 3C). Regionally, malaria burden was most severe in Western Africa and Central Africa, with significant transmission also observed in Eastern Africa. Chagas disease predominated in Australasia (100%), high-income North America (99.8%), southern Latin America (99.1%), and western Europe (91.3%), with substantial DALY contributions in Australasia, high-income North America, and southern Latin America. Additionally, Chagas disease accounted for significant mortality burdens in Latin American regions, including southern Latin America (99.8%), Andean Latin America (93.6%), Tropical Latin America (76.1%), and Central Latin America (66.9%). Leishmaniasis has emerged as the predominant disease in Central Asia and Central Europe, representing the largest proportions of cases, deaths, and DALYs in these regions. Schistosomiasis is prevalent primarily in East Asia. Lymphatic filariasis was concentrated in South Asia (84.6%), Southeast Asia (74.0%), and Oceania (53.3%) and accounted for the majority of DALYs in Southeast Asia. In 2021, African trypanosomiasis and onchocerciasis had relatively low proportion of cases across all 21 regions analyzed (Figure 3A–C).

### 3.3. Age and Sex Disparities of VBPDs

**Figure 4:**
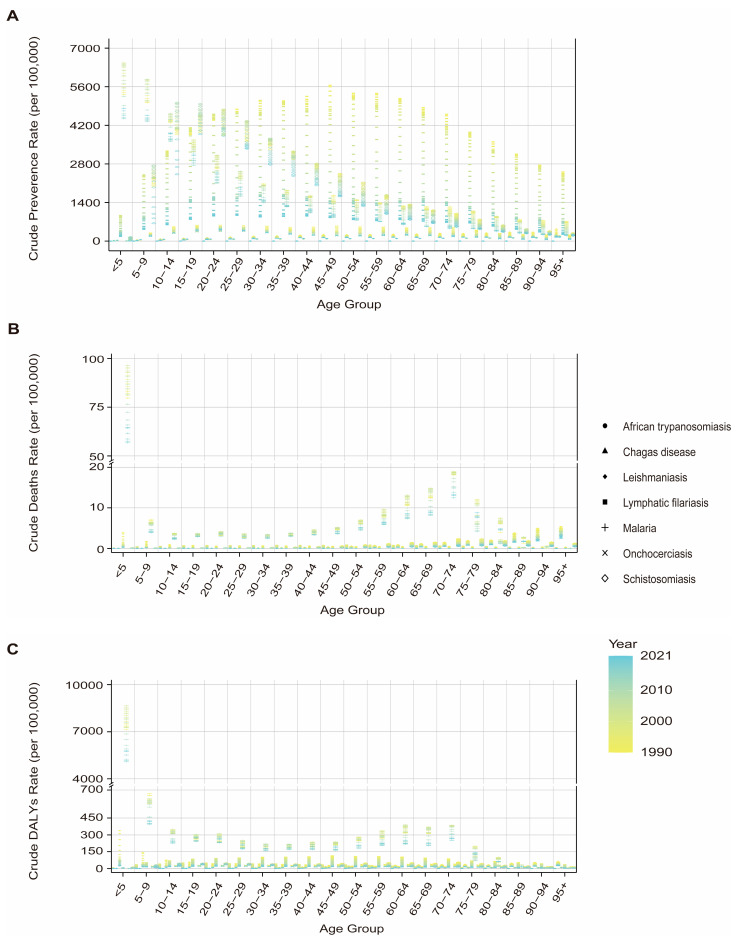
Crude prevalence (**A**), death (**B**), and DALY (**C**) rates of VBPDs in different age groups from 1990 to 2021. VBPDs, vector-borne parasitic diseases; DALY, disability-adjusted life year.

During the period from 1990 to 2021, the majority of VBPDs cases were concentrated in the 15–49 year age group, with this demographic also contributing significantly to the DALYs of malaria, African trypanosomiasis, schistosomiasis, lymphatic filariasis, and onchocerciasis (Figure 1 and Figure 2). For malaria, children under five presented relatively high prevalence rates, death counts, mortality rates, and DALY rates (Figure 1A–C and Figure 4, Tables S7–S9). Similarly to its prevalence, African trypanosomiasis deaths and DALYs attained maximum in the 15–49 age group, although the death rate reached the highest level in the 50–54 age group. Notably, the burden of disease, measured in DALYs, was elevated in children and adolescents, with the heaviest burden falling on the 15–19 year age group. (Figure 1G–I). Schistosomiasis was most prevalent in the 15–19-year-old cohort, followed by a gradual decline with age. While the most deaths were concentrated in the 50–74-year age group, the death rate peaked at approximately 75 years. Its DALY rate remained relatively low in the under 5 and over 90 years of age groups but stabilized at consistent levels across the other age groups (Figure 1D–F, Figure 4, Tables S7–S9). Lymphatic filariasis maintained a low prevalence rate under age 9, increased significantly thereafter and remained persistently high until age 70. The DALY rate was lowest in infants and young children (under 5), peaked at 40–50 years of age, and declined moderately after 65 years of age while maintaining elevated levels (Figure 4, Tables S7–S9). Onchocerciasis demonstrated a prevalence peak at 20–24 years of age, with minimal age-related variation in the DALY rate (Figure 4, Tables S7–S9). Chagas disease was associated with a concentration of deaths and DALYs in the 50–74 years age group, although the prevalence, mortality, and DALY rates all peaked among people over 95 (Figure 1J–L, Figure 4, Tables S7–S9). For leishmaniasis, the deaths and DALYs were clustered in the 15–49 year age group, whereas both the prevalence and mortality rates were highest in those over 95 years. Additionally, its DALY rate peaked in children younger than 5 years (Figure 1M–O, Figure 4, Tables S7–S9).

**Figure 5:**
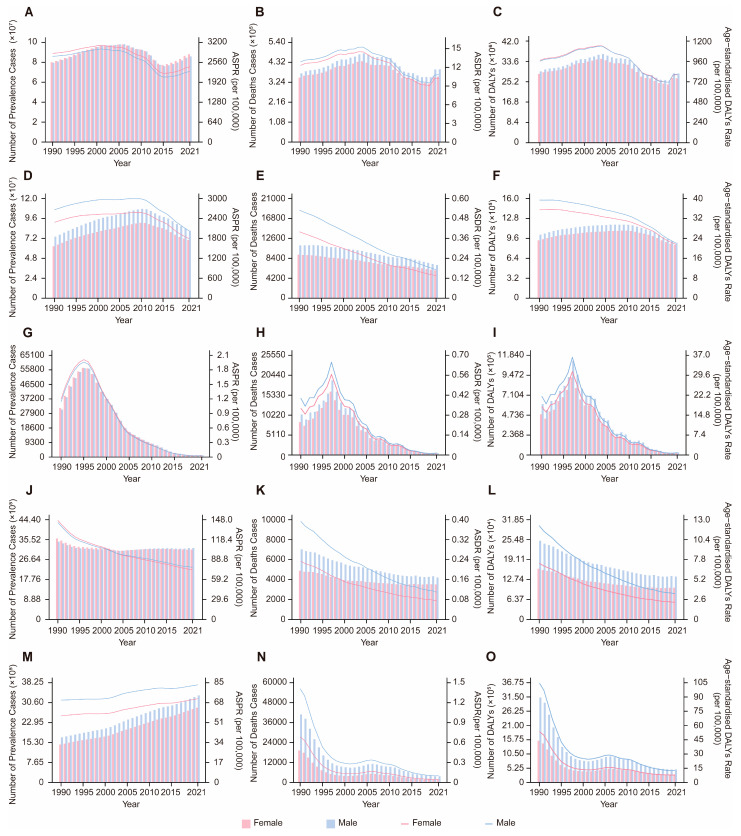
Trends in the number of prevalence, deaths, DALYs and ASPR, ASDR, age-standardized DALY rates by sex from 1990 to 2021. (**A**–**C**), malaria. (**D**–**F**), schistosomiasis. (**G**–**I**), African trypanosomiasis. (**J**–**L**), Chagas disease. (**M**–**O**), leishmaniasis. DALY, disability-adjusted life year; ASPR, age-standardized prevalence rate; ASDR, age-standardized death rate.

**Figure 6:**
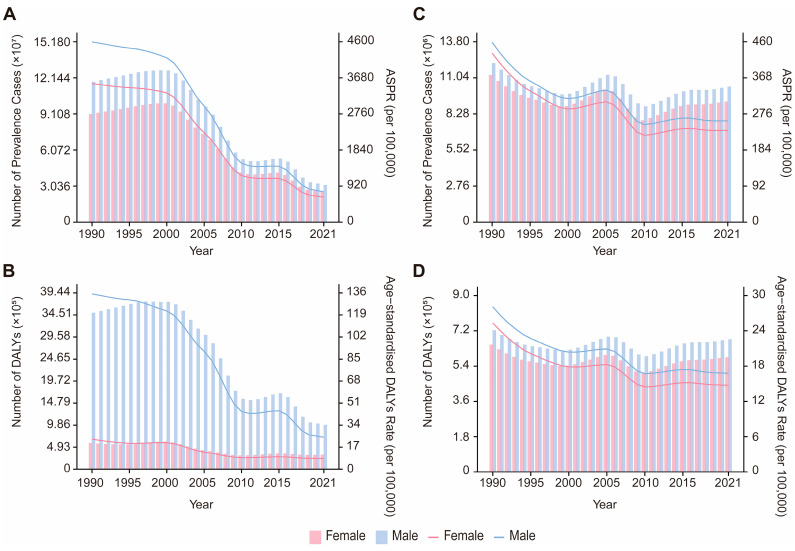
Trends in the number of prevalence, DALYs and ASPR, age-standardized DALY rates by sex from 1990 to 2021. (**A**,**B**), lymphatic filariasis. (**C**,**D**), onchocerciasis. DALY, disability-adjusted life year; ASPR, age-standardized prevalence rate; ASDR, age-standardized death rate.

Gender differences showed that the DALY burden of VBPDs was greater in males than in females. Between 1990 and 2021, with the exception of malaria, the magnitude of change in age-standardized DALY rates was greater among males compared to females (Figure 5 and Figure 6, Table S3). For malaria, the prevalence burden was lower for males relative to females, but the mortality burden was greater in males (Figure 5A–C). Additionally, from 1990 to 2021, the ASPR changed more substantially in males than in females, whereas the ASDR and age-standardized DALY rate exhibited smaller changes in males than in females (Tables S1–S3). For Chagas disease and African trypanosomiasis, sex-based differences in case numbers and prevalence rates were negligible. However, males bore a greater burden in terms of deaths and DALYs (Figure 5G–L). Notably, the male ASPR for Chagas disease changed less markedly than the female ASPR did from 1990 to 2021, whereas the opposite trend was observed for African trypanosomiasis (Table S1). Leishmaniasis and schistosomiasis consist-ently had greater burdens on males in terms of prevalence, mortality, and DALYs, with faster changes in age-standardized DALY rates among males (Figure 5D–F,M–O, Table S3). For leishmaniasis, the female ASPR and ASDR changed more rapidly, whereas the reverse was true for schistosomiasis (Tables S1 and S2). Both lymphatic filariasis and onchocerciasis presented relatively high prevalence rates and DALY burdens in males. However, lymphatic filariasis exhibited faster changes in the male ASPR and age-standardized DALY rates, whereas onchocerciasis showed the opposite pattern (Figure 6, Tables S1 and S3).

### 3.4. Associations Between the Burden of VBPDs and the SDI

**Figure 7:**
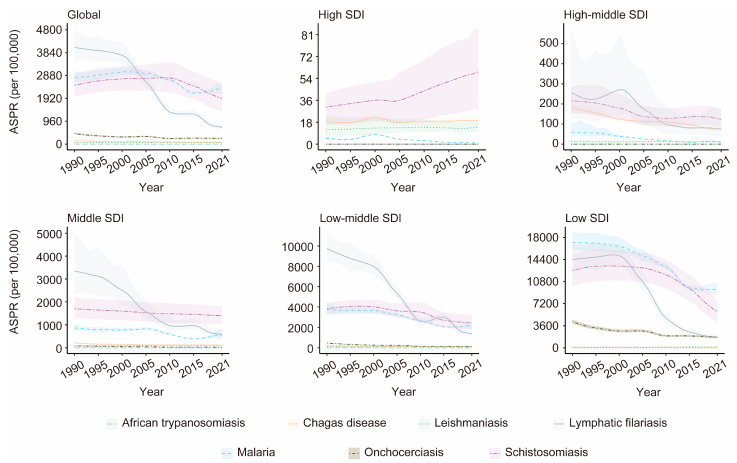
The changing trends of the ASPR for the VBPDs were analyzed among the global and five SDI regions. VBPDs, vector-borne parasitic diseases; ASPR, age-standardized prevalence rate.

**Figure 8:**
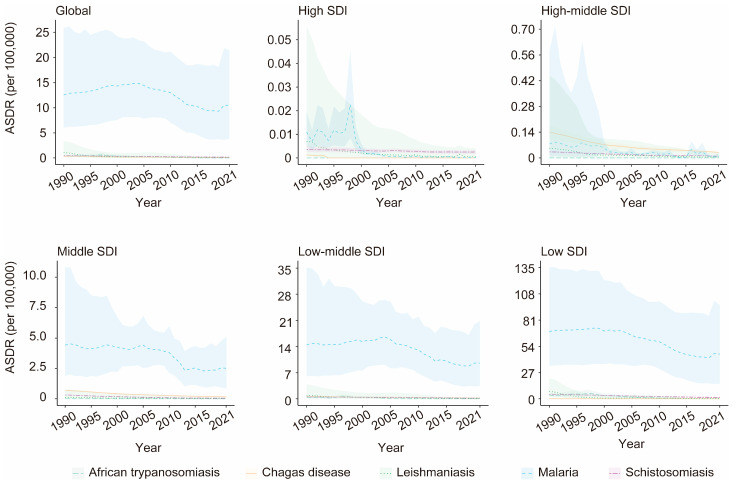
Changes in the ASDR for the VBPDs among the global and five SDI regions. VBPDs, vector-borne parasitic diseases; ASDR, age-standardized death rate.

**Figure 9:**
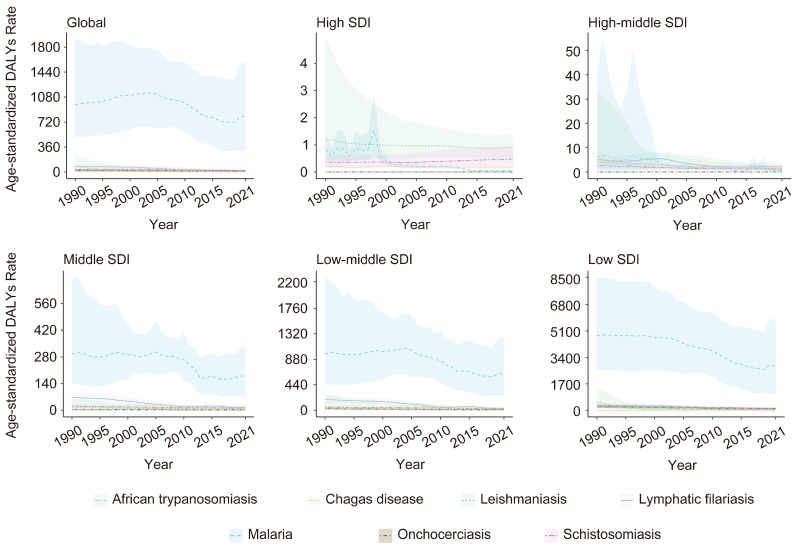
Changes in the age-standardized DALY rates for the VBPDs were analyzed among the global and five SDI regions. VBPDs, vector-borne parasitic diseases; DALY, disability-adjusted life year.

**Figure 10:**
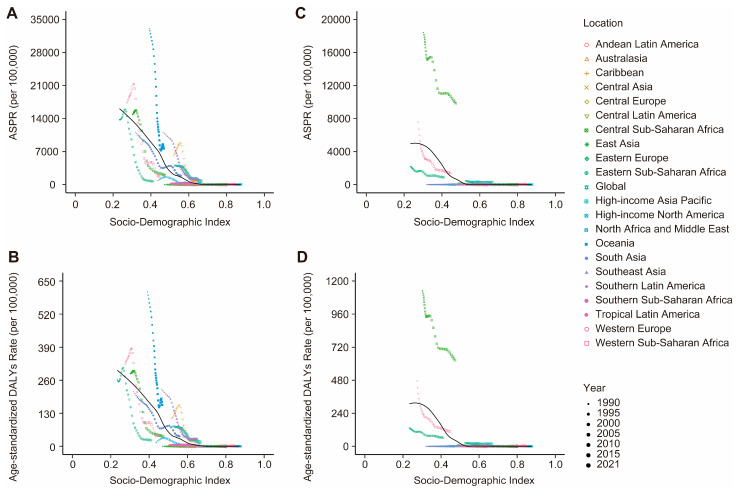
Association between the ASPR, and age-standardized DALY rate and the SDI globally and in 21 geographical regions from 1990 to 2021. (**A**,**B**), lymphatic filariasis. (**C**,**D**), onchocerciasis. ASPR, age-standardized prevalence rate; ASDR, age-standardized death rate; DALY, disability-adjusted life year; SDI, Socio-demographic Index.

**Figure 11:**
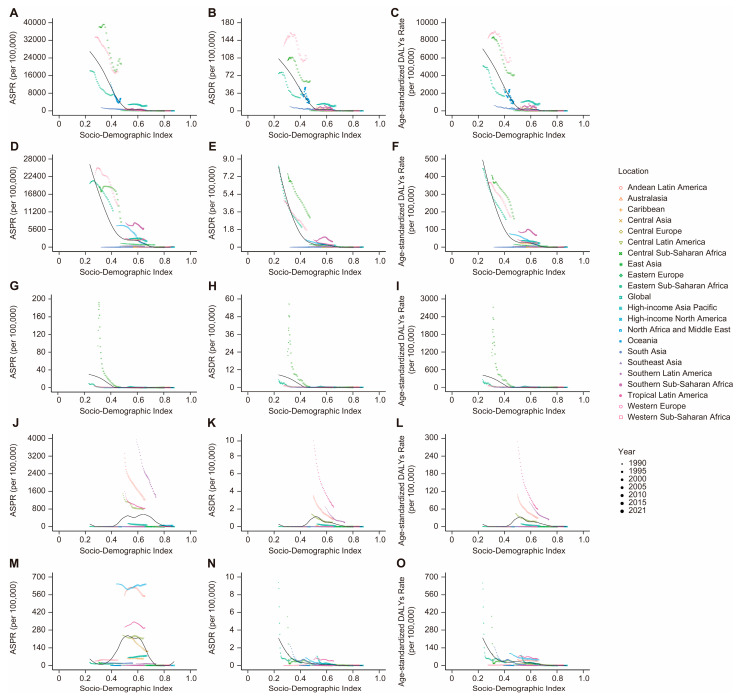
Association between the ASPR, ASDR, and age-standardized DALY rate and the SDI globally and in 21 geographical regions from 1990 to 2021. (**A**–**C**), malaria. (**D**–**F**), schistosomiasis. (**G**–**I**), African trypanosomiasis. (**J**–**L**), Chagas disease. (**M**–**O**), leishmaniasis. ASPR, age-standardized prevalence rate; ASDR, age-standardized death rate; DALYs, disability-adjusted life years; SDI, Socio-demographic Index.

From 1990 to 2021, among the SDI regions where malaria, African trypanosomiasis, lymphatic filariasis, or onchocerciasis cases were reported, the ASPR for these four diseases significantly decreased. The ASPR trend for both Chagas disease and schistosomiasis shows an overall decline, rising only slightly in high-SDI regions. In contrast, the ASPR for leishmaniasis demonstrated an overall increasing trend, with declines observed only in low-middle-SDI regions (Figure 7, Table S1). Significant declines occurred in both ASDR and age-standardized DALY rates for reported VBPDs across all SDI regions, although DALYs for schistosomiasis rose slightly in high-SDI regions (Figure 8 and Figure 9, Tables S1 and S2). With respect to lymphatic filariasis and onchocerciasis, both the ASPR and age-standardized DALY rates generally decreased as SDI increased (Figure 10, Tables S10–S17). Similarly, for African trypanosomiasis, malaria, and schistosomiasis, a negative correlation was observed between ASPR, ASDR, age-standardized DALY rates, and SDI. The most noticeable decline occurred in regions where the SDI was less than 0.4, and the trend flattened as the SDI increased. In the case of Chagas disease, the ASPR, ASDR, and age-standardized DALY rates were nonlinearly related to the SDI. The ASPR was greater when the SDI was between 0.4 and 0.6, gradually decreased after the SDI exceeded 0.6, and stabilized when the SDI surpassed 0.8. For leishmaniasis, the ASPR exhibited a nonlinear relationship with the SDI, remaining high when the SDI was between 0.5 and 0.7. However, ASDR and age-standardized DALY rates showed an inverse linear relationship with SDI, decreasing as SDI rose and plateauing once SDI surpassed 0.4. Regions with lower SDIs consistently bore higher burdens (Figure 11).

### 3.5. Forecasting the Global Burden of VBPDs with the ARIMA Model

**Figure 12:**
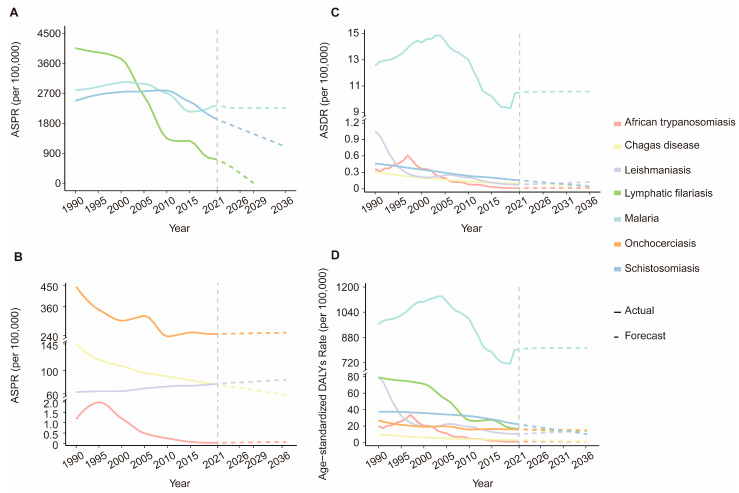
Predictions of the ASPR (**A**,**B**), ASDR (**C**) and age-standardized DALY rate (**D**) for VBPDs from 2022 to 2036 via the ARIMA model. ASPR, age-standardized prevalence rate; ASDR, age-standardized death rate; DALY, disability-adjusted life year; VBPDs, vector-borne parasitic diseases; ARIMA: autoregressive integrated moving average.

The ARIMA model was employed to forecast trends in age-standardized indicators of VBPDs over the next 15 years. The optimal model parameters, along with their corresponding AIC, BIC, and Ljung–Box test p-values, are detailed in Table S18. The Ljung–Box test confirmed that all models exhibited white noise residuals, demonstrating their stability and indicating a strong fit to the data. According to the model prediction results, the global burden of VBPDs will exhibit divergent trends over the next 15 years (Figure 12). Malaria, African trypanosomiasis, and onchocerciasis are projected to stabilize without significant fluctuations. Schistosomiasis and Chagas disease show declining trends across all three metrics, with particularly pronounced reductions in their ASPR. The ASPR of lymphatic filariasis is expected to approach near-zero levels by 2029, although its age-standardized DALYs remain largely unchanged. Notably, leishmaniasis stands out as the only disease with increasing trends in all three indicators: its ASPR surged significantly, contrasting with slight increases in ASDR and the age-standardized DALY rate.

## 4. Discussion

This study systematically reveals the burden evolution trends and distribution characteristics of VBPDs globally from 1990 to 2021. While the burden of diseases like Chagas disease and African trypanosomiasis has fallen significantly, malaria and schistosomiasis remain major public health concerns, and the rising incidence of leishmaniasis demands greater attention. Among the 21 GBD regions, Western Africa and Central Africa experienced the most severe combined burden of VBPDs, where the co-endemic transmission of malaria, African trypanosomiasis, schistosomiasis, lymphatic filariasis, and onchocerciasis is concentrated. This geographic concentration aligns with environmental determinants: VBPDs are distributed mainly in tropical and subtropical regions due to climate suitability for vectors, socioeconomic disparities, and limited healthcare access. The warm, rainy climate (typically > 20 °C with high humidity) favors the survival and reproduction of vectors like mosquitoes and sandflies [26,27,28]. These biodiverse regions provide multiple hosts and transmission routes for zoonotic VBPDs [29]. Climate change exacerbates these risks through increased heatwaves and floods, expanding vector habitats to previously unsuitable areas and intensifying pathogen development [30,31]. Environmental degradation from human activities further facilitates pathogen spread [32]. Compounding these challenges, tropical regions often have inadequate health infrastructure, poor sanitation, and agricultural practices (e.g., deforestation, irrigation) that create optimal parasite habitats [29].

Consequently, in high-burden regions like Western and Central Africa, priorities include enhancing diagnostic facilities and surveillance sensitivity to detect co-infections, expanding clean water infrastructure and sanitation to interrupt water-based transmission and integrating climate adaptation measures into vector control programs. These geographically stratified burden patterns—combined with temporal trends and demographic disparities revealed in our analysis—provide a granular evidence base for (a) rigorously evaluating progress of disease-specific control programs in priority regions (e.g., malaria reduction in Western Africa vs. leishmaniasis resurgence in Central Asia); (b) identifying critical gaps in current interventions (e.g., persistent schistosomiasis hotspots despite SDI improvements); (c) optimizing resource allocation by prioritizing high-burden regions and vulnerable populations (e.g., children under five in malaria-endemic areas). Continued global burden monitoring is essential for evidence-based policy-making and accelerating elimination of targeted VBPDs.

From the perspective of the SDI, there are notable differences in the disease burden across regions with varying SDI levels. Overall, low-SDI regions bear a heavier disease burden. Countries such as the Democratic Republic of the Congo and South Sudan, for instance, face compounded challenges from both socioeconomic vulnerabilities and environmental stressors—including recurrent flooding, drought cycles, and land degradation—which interact synergistically with lower economic development, poorer income levels, and fragile healthcare systems to amplify VBPD transmission risks. These factors contribute to deficiencies in disease prevention and treatment [33]. In medium-SDI countries, such as in Karachi, Pakistan, higher burdens of diseases such as Lymphatic filariasis and leishmaniasis are observed. This is closely linked to the environmental degradation associated with early-stage economic development, particularly accelerated urbanization, which exacerbates pressure on infrastructure and public health systems, further promoting vector-borne transmission [34]. In high-SDI regions, while most infectious diseases are effectively controlled, the increased transmission of certain diseases, such as schistosomiasis, driven by international trade and population mobility, has led to a corresponding increase in disease burden [14].

Overall, from 1990 to 2021, the global burden of VBPDs declined significantly, with particularly notable reductions in African trypanosomiasis, Chagas disease, lymphatic filariasis, and onchocerciasis. These achievements reflect the remarkable success of region-specific control measures, particularly targeted vector control and mass drug administration programs [21,35,36,37]. African trypanosomiasis exhibited the most dramatic decline, with the prevalence plummeting after its 1995 peak. By 2022, seven countries had received WHO certification for eliminating transmission of gambiense human African trypanosomiasis [38]. The key factors involved in its near-eradication ability include enhanced surveillance systems, advanced diagnostic tools, innovative therapeutics, and effective vector control strategies [39,40,41,42,43]. Notably, Uganda became the first country to eliminate the disease through large-scale screening, treatment, and deployment of “Tiny Targets”—insecticide-treated traps that drastically reduced tsetse fly populations and interrupted transmission [41]. However, despite significant progress in the control of VBPDs, malaria remains the most burdensome VBPD globally. Since 2015, there has been a resurgence in malaria cases, primarily due to increasing drug resistance in Plasmodium parasites and insecticide resistance in mosquito vectors. This has not only made malaria treatment more challenging but also reduced the protective efficacy of key vector control measures such as insecticide-treated nets and outdoor residual spraying [18,19]. In addition to insecticide resistance issues, adaptive changes in mosquito vector behavior, such as outdoor biting, also pose new threats to malaria control [20,44].

According to GBD 2021 data, the burdens of prevalence, mortality, and DALYs for most VBPDs are greater in males than in females. This may be due to occupational risks, such as men working outdoors more frequently than women and being more exposed to contaminated water or animal blood [45,46]. Notably, although the disease prevalence of malaria in women is similar to that in men, the mortality rate and DALY rate are lower in women. While experimental studies suggest potential immunomodulatory roles of hormones, including estrogen’s influence on Th1/Th2 cytokine balance and macrophage polarization [47], these mechanisms extend beyond the scope of population-the level GBD metrics. The contribution of biological factors to sex-disaggregated disease outcomes warrants further investigation through targeted immunological studies.

VBPDs’ burden differs markedly between age groups. Our findings reveal that children bear the core burden of malaria. Owing to immature immune systems, underdeveloped blood–brain barriers in young children, limited vaccine protection, and atypical malaria symptoms, the under-5 population endure the highest risk of deaths and DALY burdens. In endemic regions with high infection rates, additional risk factors, including frequent outdoor activities, low mosquito net usage, and malnutrition, amplify the risk of malaria infection in children [48]. Moreover, the DALY peak for leishmaniasis occurs in population under five, indicating that although the prevalence and mortality of leishmaniasis are lower in children than in elderly individuals, the disease causes greater health losses in children because of their weaker physiological resilience. On the other hand, terminal complications of VBPDs increase mortality risks in older adults, exemplified by conditions such as Chagas cardiomyopathy, schistosomiasis-induced hepatic fibrosis, and portal hypertension [10,15,49]. This distribution difference suggests that control strategies should be age-differentiated. For the child population, exposure interventions should be strengthened, such as increasing mosquito net coverage, whereas for elderly infected individuals, a corresponding monitoring system for complications should be established.

This study applied the ARIMA model to predict the disease burden of VBPDs over the following 15 years. The results revealed different potential trends for various diseases. For lymphatic filariasis, the ASPR is expected to approach zero in approximately 2029, which aligns with the WHO global elimination program. Although the predictions are optimistic, mass drug administration and integrated transmission-interruption strategies should be sustained. Notably, the ASPR, ASDR, and age-standardized DALY rates for leishmaniasis all increased slightly, potentially associated with climate change, human mobility, and increasing pathogen resistance. This underscores the need to strengthen epidemiological monitoring and pathogen containment. Furthermore, understanding the role of these factors in driving disease trends is crucial for developing future intervention strategies.

Using the GBD 2021 database, we analyzed the global, regional, and national disease burden and trends of VBPDs from 1990 to 2021. By integrating the EAPC and ARIMA models, the dynamic changes in diseases were comprehensively revealed, and future trends were predicted. However, limitations exist in this research. First, GBD estimates are contingent upon existing data sources, and health data from some low- and middle-income countries—particularly in certain regions of Africa and Asia—may lack completeness and accuracy, potentially introducing estimation biases [50]. Second, the ARIMA model itself has inherent constraints. The ARIMA model requires substantial data volumes, yet information delays in the database may compromise prediction accuracy [51]. Additionally, as the ARIMA model primarily builds on the intrinsic dynamic characteristics of time series data, it faces difficulty in directly incorporating external factors that influence VBPDs, such as climate change, social development factors, and public health policies.

## 5. Conclusions

These findings indicate that VBPDs, particularly malaria and schistosomiasis, remain persistent public health threats. The projected rise in leishmaniasis prevalence over the coming 15 years necessitates increased vector surveillance and control efforts. Geographically, sub-Saharan Africa bears the heaviest integrated burden, reflecting the compounding effects of environmental conditions, socioeconomic vulnerabilities, and infrastructure deficiencies on VBPD transmission. Furthermore, low-SDI regions endure disproportionately higher burdens owing to limited healthcare resources, environmental degradation, and inadequate vector control measures. By analyzing regional and demographic disparities in disease distribution, this study provides critical epidemiological evidence for developing optimized intervention strategies. In low-SDI regions, priorities should include strengthening basic health infrastructure, scaling up vector control programs, and leveraging international assistance to address multiple disease burdens. Middle-SDI regions require balanced approaches that integrate urbanization management with ecological conservation alongside intersectoral collaboration and antimicrobial resistance monitoring. High-SDI regions should focus on border screening for imported cases, technological innovation, and public health education. Gender- and age-specific strategies are essential: occupational exposure prevention for males, vaccination and nutritional interventions for children, and screening for complications in elderly populations.

In summary, this systematic analysis of the global VBPDs burden from 1990 to 2021 highlights both the progress achieved and the persistent challenges in disease control. Addressing these disparities through targeted interventions will be crucial for achieving elimination targets and advancing global health equity. The insights gained emphasize the necessity of sustained commitment to surveillance systems, resource allocation optimization, and tailored strategies responsive to regional socioeconomic contexts and population characteristics.

## Data Availability

Data on these VBPDs can be accessed through the Global Health Data Exchange (GHDx) Results Tool (http://ghdx.healthdata.org/gbd-results-tool, accessed on 6 December 2024) and can be obtained through a web browser on its official website.

## Supplementary Materials

The following supporting information can be downloaded at: https://www.mdpi.com/article/10.3390/pathogens14090844/s1, Table S1: The ASPR of VBPDs in 2021, and the EAPC of ASPR from 1990 to 2021; Table S2: The ASDR of VBPDs in 2021, and the EAPC of ASDR from 1990 to 2021; Table S3: The age-standardized DALY rate of VBPDs in 2021, and the EAPC from 1990 to 2021; Table S4: Number and proportion of prevalence cases of VBPDs by region in 2021; Table S5: Number and proportion of death cases of VBPDs by region in 2021; Table S6 Number and proportion of DALYS of VBPDs by region in 2021; Table S7: The crude prevalence rate of VBPDs in 1990 and 2021 by age; Table S8: The crude deaths rate of VBPDs in 1990 and 2021 by age; Table S9: The crude DALYs rate of VBPDs in 1990 and 2021 by age; Table S10: Socio-demographic Index values for 21 GBD 2021 regions, 1990–2021; Table S11: The ASPR, ASDR and age-standardized DALYs rate of malaria from 1990 to 2021 by SDI regions; Table S12: The ASPR, ASDR and age-standardized DALYs rate of schistosomiasis from 1990 to 2021 by SDI regions; Table S13: The ASPR, ASDR and age-standardized DALYs rate of African trypanosomiasis from 1990 to 2021 by SDI regions; Table S14: The ASPR, ASDR and age-standardized DALYs rate of Chagas disease from 1990 to 2021 by SDI regions; Table S15: The ASPR, ASDR and age-standardized DALYs rate of leishmaniasis from 1990 to 2021 by SDI regions; Table S16: The ASPR, ASDR and age-standardized DALYs rate of Lymphatic filariasis from 1990 to 2021 by SDI regions; Table S17: The ASPR, ASDR and age-standardized DALYs rate of Onchocerciasis from 1990 to 2021 by SDI regions; Table S18: ARIMR model parameters and their corresponding AIC, BIC and Ljung–Box test *p* value.

## Abbreviations

The following abbreviations are used in this manuscript:

VBPDs	Vector-borne parasitic diseases
GBD	Global Burden of Diseases
DALYs	Disability-adjusted life years
SDI	Socio-demographic Index
EAPC	Estimated annual percentage change
ARIMA	Autoregressive integrated moving average
AIC	Akaike Information Criterion
BIC	Bayesian Information Criterion
WHO	World Health Organization
ASR	Age-standardized rate
ASPR	Age-standardized prevalence rate
ASDR	Age-standardized death rate
CI	Confidence interval
